# Empagliflozin Alleviates Osteoarthritis Progression by Attenuating Inflammation, Restoring Impaired Autophagy, and Ameliorating Chondrocyte Senescence

**DOI:** 10.3390/biomedicines14040828

**Published:** 2026-04-05

**Authors:** Junhong Li, Guihua Yu, Shiheng Wang, Zekai Zhang, Yu Wen, Luting Yu, Xin Gan, Hao Kang, Jinming Zhang, Lu He

**Affiliations:** 1 Department of Pediatrics, Tongji Hospital, Tongji Medical College, Huazhong University of Science and Technology, Wuhan 430030, China; 2 Department of Orthopedics, Tongji Hospital, Tongji Medical College, Huazhong University of Science and Technology, Wuhan 430030, China; 3 Department of Orthopedics, Ezhou Central Hospital, Ezhou 436000, China

**Keywords:** empagliflozin, osteoarthritis, cartilage, inflammation, senescence, autophagy

## Abstract

**Background:** Osteoarthritis (OA) is a multifactorial disease, including inflammation, autophagy and senescence. Published work has indicated that empagliflozin (EMP) exhibits robust anti-inflammatory and anti-senescence effects, while its role in autophagy appears paradoxical. Here, we aim to identify the chondroprotective effect of EMP on OA. **Methods:** An OA model was established both in vitro, by stimulating primary chondrocytes (isolated from C57BL/6J mice) with IL-1β, and in vivo, by performing (Destabilized medial meniscus) DMM surgery on C57BL/6J mice. (Western blot) WB and (quantitative real-time polymerase chain reaction) qRT-PCR analysis were employed to detect the gene expression. (Immunofluorescence) IF staining was employed to detect the expression and location of target protein. SA-β-gal staining was employed to evaluate cellular senescence. Autophagic flux was assessed using a GFP-RFP-LC3 adenoviral vector. Network pharmacology was applied to identify potential pathways for experimental validation. The effects of EMP in vivo were evaluated by μ-CT, histological and (Immunohistochemistry) IHC staining. **Results:** EMP promoted anabolism, inhibited the inflammatory response and catabolism in IL-1β stimulated chondrocytes. EMP enhanced autophagic activity and attenuated senescent phenotype in vitro. Mechanistically, EMP regulated the PI3K/Akt/mTOR and AMPK pathways. The chondroprotective effects of EMP were reversed by (3-methyladenine) 3-MA. EMP also ameliorated OA-related phenotype in DMM models. Compared with (Kartogenin) KGN, EMP showed more pronounced suppression of inflammatory and catabolic markers, while both compounds similarly promoted anabolic marker expression. **Conclusions:** These in vitro and in vivo data collectively indicates that EMP can alleviate OA both in IL-1β stimulated chondrocytes and DMM induced models. Beyond its established role in diabetes management, EMP is evaluated in the context of OA, emerging as a novel and promising therapeutic agent for OA.

## 1. Introduction

Osteoarthritis (OA) is recognized as the most prevalent joint disorder worldwide [1]. An estimated 10% of men and 18% of women aged over 60 are affected globally, constituting approximately 7% of the world’s population in 2020 [2]. With aging demographics, an exponential increase in this value was projected to occur by the year 2050, posing a substantial and growing socioeconomic burden [3]. OA is a primary contributor to debilitating sequelae, including chronic pain, disability, and diminished quality of life, particularly in the elderly [4]. Despite the high prevalence of OA, no curative and effective treatments have been established for this disease. In clinical practice, current OA management strategies are primarily palliative. In early to moderate stage OA, pain is managed pharmacologically, including locally applied and systemically delivered analgesic agents [3,4]. While for advanced stage OA, joint replacement is indicated [5]. However, pharmacological interventions are largely aimed at short-term symptom relief or functional recovery rather than altering disease progression [4]. Besides, arthroplasty is associated with limitations such as suboptimal functional recovery in some patients, increased economic burden and a finite prosthesis lifespan [6,7]. Consequently, given the significant socioeconomic burden imposed by OA and the limitations of existing therapies, the development of novel pharmaceutical agents capable of slowing or halting disease progression is considered a crucial clinical objective.

OA pathogenesis involves a complex interplay between inflammation, autophagy dysregulation, and cellular senescence [8]. Autophagy modulates a spectrum of physiological processes, including cellular senescence and death [9,10,11]. Inflammation can not only alter the catabolism and anabolism in chondrocytes, but also alter the autophagy process and senescence state. Pro-inflammatory conditions suppress autophagic flux and induce senescence-associated markers in chondrocytes [12]. Dysfunction of the autophagic process also triggers cellular senescence [13]. Moreover, cellular senescence, in turn, results in chronic inflammation: senescent cells could release proinflammatory cytokines and extracellular matrix (ECM)-degrading enzymes, further alter tissue microenvironments and contribute to the disruption of cartilaginous tissue homeostasis [1,14]. Therefore, improved understanding of the important role of inflammation- autophagy-senescence network in OA pathogenesis is considered promising for OA treatment. Novel treatment strategies targeting the inflammation-autophagy-senescence network may slow or stop OA progression.

Empagliflozin (EMP) is a sodium-glucose cotransporter-2 (SGLT2) inhibitor and widely utilized in clinical practice for glycemic management [15]. In addition to its established hypoglycemic effects, EMP showed a range of biological activities. Firstly, EMP exhibited potent anti-inflammatory effects in multiple conditions [16,17,18], For example, Kim et al. demonstrated that EMP inhibited the activation of astrocyte and inflammation induced by high-fat diet through regulation of the NF-κB pathway [17]. Yang et al. revealed that EMP could attenuate inflammation in renal ischemia-reperfusion injury by regulating the AMPK pathway [18]. Secondly, EMP exhibited paradoxical role in autophagy. Autophagy improvements following EMP treatment were observed in some studies [19,20,21,22]. While other studies reported the inhibition of autophagy following EMP treatment [23]. Thirdly, several studies have also shown the beneficial effects of EMP in terms of senescence [24,25]. However, the role of EMP in OA, particularly in chondrocytes, remains largely undefined. Based on this, we hypothesized that EMP exerts chondroprotective effects in OA by attenuating inflammation, restoring autophagy, and suppressing cellular senescence in chondrocytes.

In this study, we aim to experimentally investigate the chondroprotective effects of EMP on OA in vivo and in vitro, which, to the best of our knowledge, have not been previously reported. A comprehensive evaluation was conducted to investigate the impact of EMP on chondrocytes, with a focus on intracellular metabolism, autophagic activity, and cellular senescence. Network pharmacology was applied to identify candidate signaling pathways, which were subsequently validated through experimental studies. Furthermore, the chondroprotective efficacy of EMP was compared with that of kartogenin (KGN).

## 2. Materials and Methods

### 2.1. Main Reagents

EMP (HY-15409) and 3-methyladenine (3-MA, HY-19312) were purchased from MedChemExpress (Monmouth Junction, NJ, USA). IL-1β was purchased from ABclonal (Wuhan, China). Fetal bovine serum (FBS) was purchased from Gibco (Waltham, MA, USA). Protein marker (MP102-01, 180 kD), SYBR qPCR Master Mix (Q713-02), and PVDF Membrane (E801-01) were obtained from Vazyme (Nanjing, China). The Cell Counting Kit-8 (CCK-8) was purchased from Boster (Wuhan, China). All primary antibodies used in this study are listed in Supplementary Table S1.

### 2.2. The Isolation and Identification of Chondrocytes

**Figure 1:**
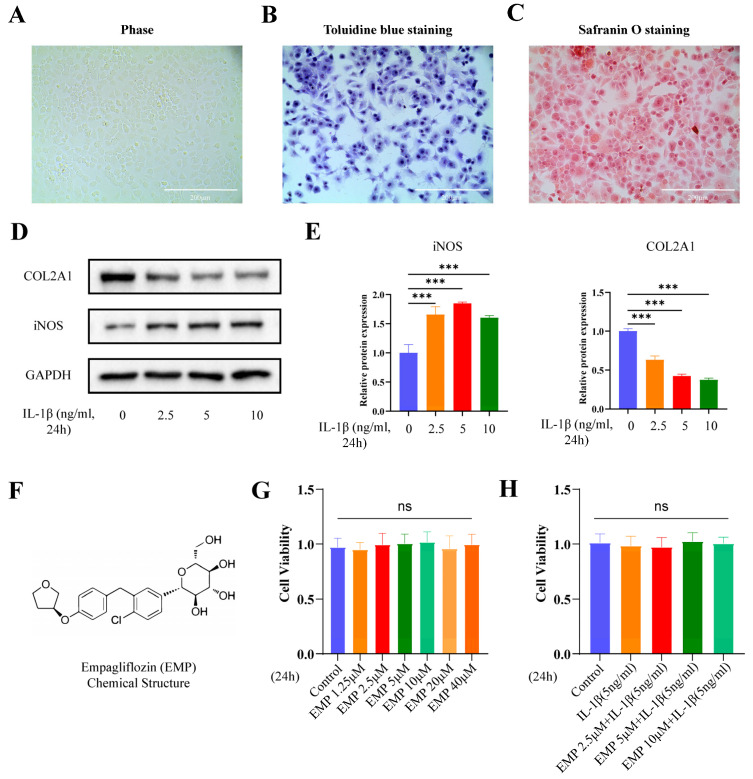
Identification of primary chondrocytes and the selection of optimal concentrations for IL-1β and EMP. (**A**) The general shape and characteristic morphology of primary chondrocytes under microscopy. (**B**) Toluidine blue staining and (**C**) Safranin O staining. (**D**) WB analyzed the protein expression level of COL2A1 and iNOS, and quantitative analysis (**E**). (**F**) The chemical structure of EMP. (**G**) The CCK8 analysis of chondrocytes viability treated with different concentrations of EMP alone or (**H**) in combination with IL-1β. Data are presented as mean ± SD (n = 3 independent biological replicates). Statistical significance was defined as *** *p* < 0.001. Non-significant differences were denoted as ns.

Chondrocytes were isolated from 5-day-old C57BL/6J mice supplied by the Experimental Animal Center of Tongji Medical College (Huazhong University of Science and Technology, Wuhan, China). Articular cartilage tissues were harvested from murine knee joints under aseptic conditions. The cartilage was minced into fragments measuring approximately 1 mm^3^. Sequential enzymatic digestion was performed using 0.25% trypsin for 30 min followed by 0.25% type II collagenase for 5 h at 37 °C. The resulting cell suspension was centrifuged at 1500 rpm for 5 min. Chondrocytes were resuspended in DMEM/F12 (1:1) medium supplemented with 10% FBS. The primary chondrocytes were identified based on their characteristic morphology under microscopy (Figure 1A) and verified by positive staining with safranin O (Figure 1B) and toluidine blue (Figure 1C). To avoid the loss of the chondrocyte phenotype, only primary or first-passage chondrocytes were used for in vitro experiments.

### 2.3. CCK-8 Assay

The effect of EMP on chondrocyte viability was assessed by CCK-8 assay. Briefly, murine chondrocytes were plated in 96-well plates at a density of 1 × 10^4^ cells per well (n = 5 replicates). Following a 24-h treatment period according to the scheme in Figure 1G,H, the culture medium was replaced with 100 μL fresh DMEM/F12 medium containing 10 μL CCK-8 reagent (Boster, Wuhan, China). After a 1-h incubation, the absorbance at 450 nm was measured using a Bio-Rad microplate reader (Hercules, Carlsbad, CA, USA).

### 2.4. Safranin O and Toluidine Blue Staining

Chondrocyte anabolic and catabolic activities as well as cellular morphology were detected via safranin O and toluidine blue staining, based on its positive correlation with proteoglycan content. Primary chondrocytes were plated in 12-well plates, and subjected to a 24-h treatment of IL-1β with or without EMP. The medium was then removed, and the cells were fixed with 4% paraformaldehyde for 15 min followed by a 30-min incubation at room temperature with the two working solutions, respectively. Stained cells were finally imaged using an Evosfl auto microscope (Life Technologies, Carlsbad, CA, USA).

### 2.5. Quantitative Reverse Transcription Polymerase Chain Reaction (qRT-PCR)

mRNA expression levels of key factors were analyzed via qRT-PCR. Chondrocytes were plated in 6-well plates and treated for 24 h. Total RNA was subsequently extracted with TRIzol reagent (Invitrogen, California, USA) according to the manufacturer’s instructions. Reverse transcription was performed to generate complementary DNA. Quantitative PCR was then carried out using the SYBR qPCR Master Mix. The primer sequences used for qRT-PCR are listed in Supplementary Table S2.

### 2.6. WB Analysis

Protein expression levels of key factors were analyzed via WB analysis. Chondrocytes were seeded in six-well plates and treated for 24 h (IL-1β or EMP). Cellular proteins were harvested using RIPA lysis buffer supplemented with 1% protease inhibitor and 1% phosphatase inhibitor. Total protein concentration was determined with a bicinchoninic acid assay kit (Boster, Wuhan, China). Equal protein aliquots (15 μg) were separated by 10% or 12% SDS-PAGE and transferred onto PVDF membranes. The membranes were blocked with 5% bovine serum albumin (BSA) for 1.5 h and subsequently incubated overnight at 4 °C with specific primary antibodies. After three 5-min washes with TBST, the membranes were probed with HRP-conjugated secondary antibodies for 1 h at room temperature. Protein bands were visualized with an enhanced chemiluminescence reagent (Boster, Wuhan, China) and imaged using a Bio-Rad Image Lab system (v5.0). All experiments were performed in triplicate. GAPDH served as the internal control.

### 2.7. Immunofluorescence (IF) Staining

Chondrocytes were plated in 48-well plates (1 × 10^4^ cells/well) and treated for 24 h. Subsequently, the cells were fixed with 4% paraformaldehyde for 30 min and permeabilized with 0.2% Triton X-100 (beyotime, Shanghai, China) for 10 min. After blocking with 1% BSA for 1 h, the cells were sequentially incubated overnight at 4 °C with primary antibodies. Following this, incubation with FITC- or Cy3-conjugated secondary antibodies (1:100 dilution) was conducted for 1 h in the dark. Nuclei were counterstained with DAPI, and fluorescent images were acquired using a fluorescence microscope (Evosfl auto microscope, Life Technologies, California, USA).

### 2.8. Senescence-Associated β-Galactosidase (SA-β-Gal) Staining

Employing an SA-β-gal staining kit (Beyotime, Haimen, China), cellular senescence was evaluated. Briefly, the treated cells were fixed with 4% paraformaldehyde and subsequently incubated in staining solution for a minimum of 16 h at 37 °C. Positively stained cells were enumerated in random microscopic fields (Nikon, Tokyo, Japan), and the positive rate was analyzed from triplicate experiments.

### 2.9. GFP-RFP-LC3 Adenovirus Transfection

Autophagic flux was assessed in chondrocytes using a GFP-RFP-LC3 adenoviral vector (HanBio Technology, Shanghai, China). Following transfection, the cells were exposed to IL-1β or EMP for a period of 24 h. After that, cells were fixed with 4% paraformaldehyde for 15 min. Puncta were visualized by a confocal microscopy (Leica, Wetzlar, Germany). Autophagosomes were identified as yellow puncta, autolysosomes were identified as red-only puncta. The number of puncta per cell was quantified using ImageJ 1.x software (Bethesda, MD, USA).

### 2.10. Network Pharmacology

Network pharmacology was applied to identify the potential targets or underlying pathways of EMP in the treatment of OA. EMP-related targets were obtained from the SwissTargetPrediction database, the Similarity Ensemble Approach database, and TargetNet database. OA-related targets were obtained from GeneCards and DisGeNET databases. In this study, a threshold was defined during target selection to enhance data validity. A total of 100 targets were retrieved from the Swiss Target Prediction database. Using a probability greater than 0 as the criterion, 17 candidate targets were identified. The Similarity Ensemble Approach (SEA) database yielded 12 relevant human targets. Additionally, 52 human targets with a probability >0 were screened from the TargetNet database. After removing duplicates across the three databases, a total of 60 targets were collected. Functional enrichment analyses, including Gene Ontology (GO) and Kyoto Encyclopedia of Genes and Genomes (KEGG) analyses, were further conducted. The functions of the intersecting target genes were annotated and described using GO analysis, with categorization based on biological process (BP), cellular component (CC), and molecular function (MF). Each category presented the top 10 significant entries. To identify the possible biological functions of EMP on OA, the KEGG database was employed for pathway analysis of the intersecting target genes. The top 30 significant pathways were showed in the bubble plot.

### 2.11. Animal Experiments

An in vivo OA model was established in male C57BL/6 mice using the destabilized medial meniscus (DMM) method, as previously described [26]. 7-week-old mice (N = 30) were randomly divided into 5 groups (six mice in each group): SHAM, DMM, DMM + KGN (20 μg/kg), DMM + EMP (5 μg/kg) and DMM + EMP (10 μg/kg). The SHAM group underwent capsulotomy only on the right knee, whereas other groups received full DMM surgery. After a 7-day recovery period, mice in the DMM + KGN (20 μg/kg), DMM + EMP (5 μg/kg) and DMM + EMP (10 μg/kg) groups were subjected to intra-articular injections of KGN or EMP (10 μL) twice weekly for eight weeks. EMP or KGN was dissolved in vehicle (10% DMSO + 40% Polyethylene glycol 300 + 5% Tween 80 + 45% Saline. The SHAM and DMM groups were administered a vehicle control on the same schedule. All animal procedures were approved by the Animal Ethics Committee of Tongji Medical College, Huazhong University of Science and Technology (IACUC Number: 4714).

### 2.12. Micro-CT, Histological and Immunohistochemical (IHC) Staining

Eight weeks post-intra-articular injection, the right knee joints were harvested and scanned by a high-resolution micro-computed tomography (μ-CT, Scanco Medical, Switzerland) at 100 kV and 98 μA, achieving a resolution of 10.5 μm. The region of interest (ROI) was defined in the tibial subchondral bone of the medial compartment, where OA-related changes are most prominent in the DMM model. Key parameters, including bone volume/tissue volume (BV/TV), trabecular separation (Tb.Sp), trabecular number (Tb.N) and trabecular thickness (Tb.Th), were quantified to evaluate structural changes in the tibial subchondral bone.

Subsequent to μ-CT scanning, the knee joints were decalcified, paraffin-embedded, and sectioned into 5 μm slices. Consecutive sections were stained with H&E, toluidine blue, and safranin O/fast green. Cartilage damage was graded using the Osteoarthritis Research Society International (OARSI) system [27]. The expression of anabolic marker (aggrecan), catabolic marker (MMP13), autophagic marker (P62), and senescence marker (P16) in vivo were assessed by immunohistochemistry (IHC) staining. All histological and IHC evaluations were performed by two independent researchers (J.L. and G.Y.) who were blinded to the experimental groups. The proportion of positively stained chondrocytes was calculated and statistically analyzed.

### 2.13. Statistical Analyses

GraphPad Prism (version 10) was employed for all statistical analyses. The normality of the data was assessed using the Shapiro-Wilk test. Variables with a normal distribution are presented as mean ± standard deviation (SD), whereas those with a non-normal distribution are expressed as median with interquartile range. Intergroup comparisons were conducted using either an unpaired Student’s t-test for parametric data or the Mann-Whitney U test for non-parametric data. For multiple group comparisons, one-way ANOVA coupled with Tukey’s post hoc test was used for parametric analysis, while the Kruskal-Wallis test followed by Dunn’s correction was applied for non-parametric analysis. Chi-square or Fisher’s exact test was adopted to analyze categorical data. A *p*-value of less than 0.05 was deemed to indicate statistical significance.

## 3. Results

### 3.1. Effect of EMP on Chondrocyte Viability

The optimal concentration for IL-1β was first selected. As shown in Figure 1D, a 24-h treatment with 5 ng/mL IL-1β significantly upregulated the protein expression level of iNOS and downregulated the protein expression level of COL2A1. Therefore, 5 ng/mL IL-1β was selected as the optimal stimulus. Based on data in Figure 1G,H demonstrating no adverse effect on chondrocyte viability by EMP (0–40 μM), either alone or with IL-1β, concentrations of 2.5, 5, and 10 μM were selected for subsequent experiments.

### 3.2. Effects of EMP on Inflammatory and Catabolic Responses in IL-1β-Stimulated Chondrocytes

**Figure 2:**
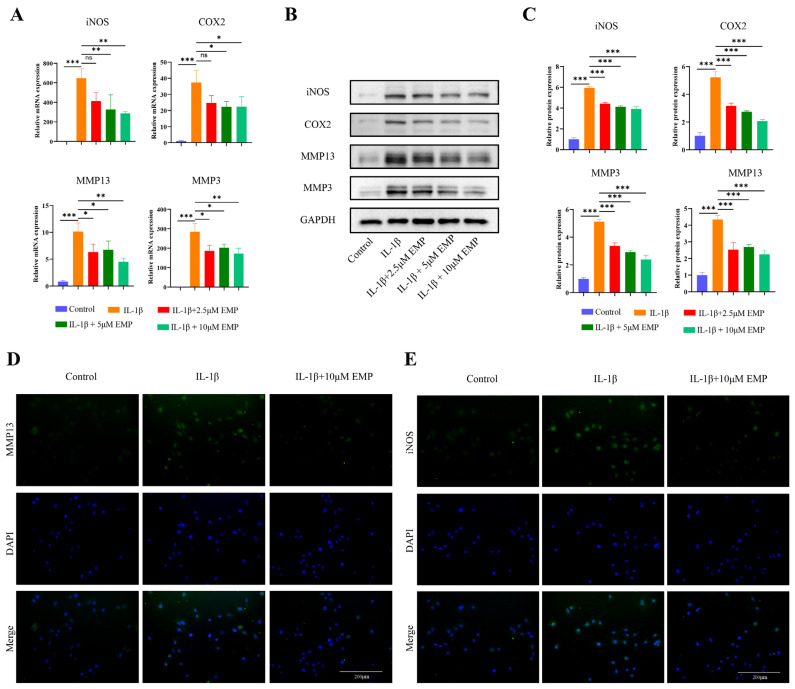
EMP inhibited the inflammatory response and catabolism in IL-1β stimulated chondrocytes. (**A**) The mRNA expression levels of iNOS, COX2, MMP13, and MMP3 were analyzed by qRT-PCR. (**B**) The protein expression levels of iNOS, COX2, MMP13, and MMP3 were analyzed by WB, and quantitative analysis (**C**). (**D**) IF staining of MMP13 and (**E**) iNOS. Data are presented as mean ± SD (n = 3). Statistical significance was defined as *** *p* < 0.001, ** *p* < 0.01 and * *p* < 0.05. Non-significant differences were denoted as ns.

As shown in Figure 2A, qRT-PCR revealed that IL-1β significantly upregulated key catabolic factors (MMP3 and MMP13) and inflammatory factors (iNOS and COX2), an effect that was reversed by co-treatment with EMP (2.5, 5 and 10 μM). The results of the WB analysis were consistent with the mRNA-level data (Figure 2B,C). This protective effect of EMP was further corroborated by IF staining, which showed markedly increased intensity of MMP13 and iNOS in the IL-1β group compared to control group, a trend that was rescued by 10 μM EMP (Figure 2D,E).

### 3.3. Effects of EMP on Anabolic Markers in IL-1β-Stimulated Chondrocytes

**Figure 3:**
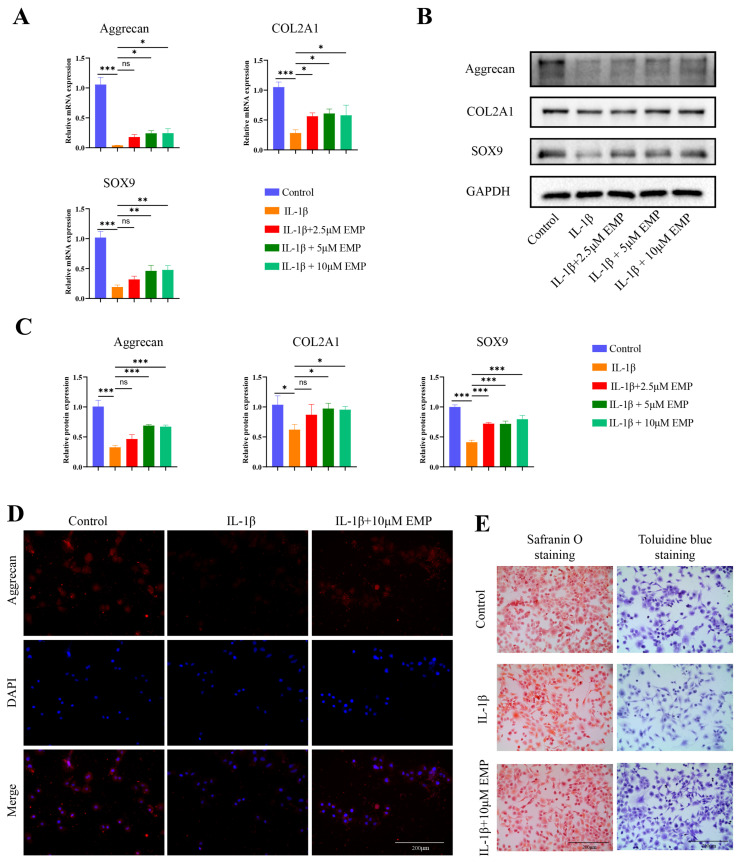
EMP promoted anabolism in IL-1β stimulated chondrocytes. (**A**) The mRNA expression levels of aggrecan, COL2A1 and SOX9 were analyzed by qRT-PCR. (**B**) The protein expression levels of aggrecan, COL2A1 and SOX9 were analyzed by WB, and (**C**) quantitative analysis. (**D**) IF staining of aggrecan. (**E**) Toluidine blue staining and Safranin O staining. Data are presented as mean ± SD (n = 3). Statistical significance was defined as *** *p* < 0.001, ** *p* < 0.01 and * *p* < 0.05. Non-significant differences were denoted as ns.

As shown in Figure 3A, qRT-PCR revealed that IL-1β significantly downregulated the mRNA expression level of key anabolic factors (aggrecan, COL2A1, and SOX9), an effect that was reversed by co-treatment with EMP (2.5, 5, and 10 μM) for 24 h. The findings of the qRT-PCR were corroborated by the WB analysis (Figure 3B,C). Consistent results were also obtained from IF staining of aggrecan (Figure 3D). Furthermore, consistent results were obtained from both safranin O and toluidine blue staining of chondrocytes (Figure 3E).

### 3.4. Comparison of the Protective Effects Between EMP (10 μM) and KGN (10 μM) in IL-1β-Stimulated Chondrocytes

**Figure 4:**
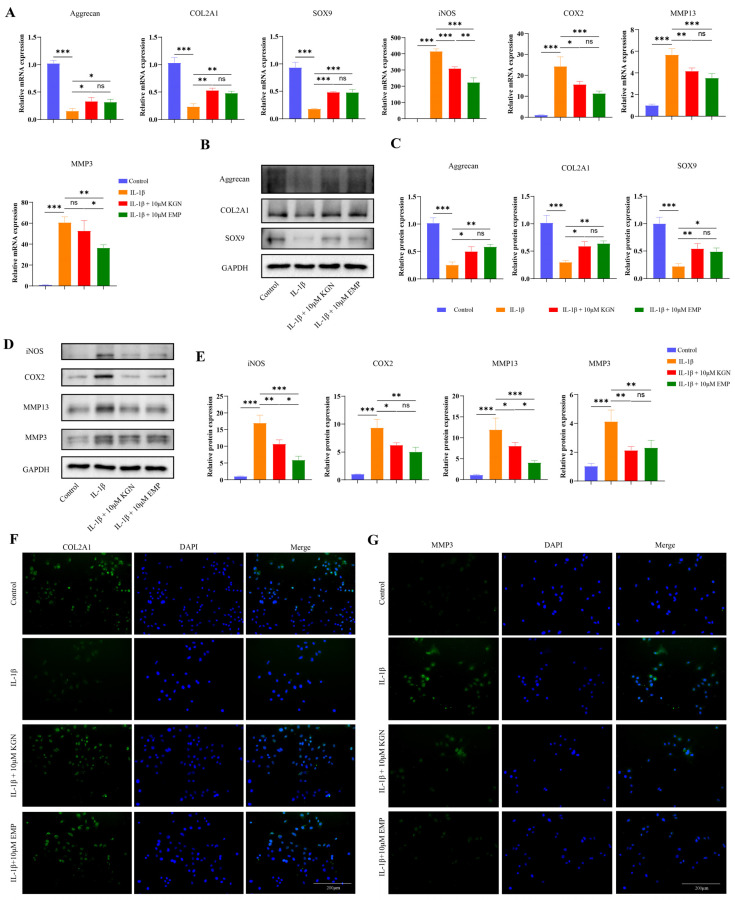
EMP (10 μM) may exhibit a potentially greater protective effect than KGN (10 μM) in IL-1β stimulated chondrocytes. (**A**) The mRNA expression levels of aggrecan, COL2A1, SOX9, iNOS, COX2, MMP13, and MMP3 were analyzed by qRT-PCR. (**B**) The protein expression levels of aggrecan, COL2A1 and SOX9 were analyzed by WB, and (**C**) quantitative analysis. (**D**) The protein expression levels of iNOS, COX2, MMP13, and MMP3 were analyzed by WB, and quantitative analysis (**E**). (**F**) IF staining of COL2A1 and MMP3 (**G**). Data are presented as mean ± SD (n = 3). Statistical significance was defined as *** *p* < 0.001, ** *p* < 0.01 and * *p* < 0.05. Non-significant differences were denoted as ns.

Based on previous work demonstrating that KGN (0.1–40 μM) showed favorable protective effect either on IL-1β stimulated chondrocytes or OA mouse model established by DMM surgery [28,29], KGN (10 μM) was selected as the positive control in the present study. The protective effects of EMP and KGN in vitro were compared by WB analysis, qRT-PCR and IF staining. Consistent with previous findings, qRT-PCR demonstrated that IL-1β downregulated the mRNA expression level of anabolic factors, and upregulated the mRNA expression level of catabolic factors and inflammatory factors. In contrast, EMP (10 μM) or KGN (10 μM) treatment for 24 h significantly upregulated the mRNA expression level of anabolic factors, and downregulated the mRNA expression level of catabolic factors and inflammatory factors in IL-1β treated chondrocytes (Figure 4A). However, EMP (10 μM) was found to possess greater chondroprotective efficacy than KGN (10 μM), specifically in terms of its impact on inflammatory and catabolic factors. For instance, the IL-1β-induced upregulation of iNOS and MMP3 was more remarkably suppressed by EMP than KGN (Figure 4A). Consistently, the results of WB analysis showed the similar trend at the protein level (Figure 4B–E). IF staining of COL2A1 and MMP3 further validated these results (Figure 4F,G).

### 3.5. Effects of EMP on Autophagy and Senescence in IL-1β-Treated Chondrocytes

**Figure 5:**
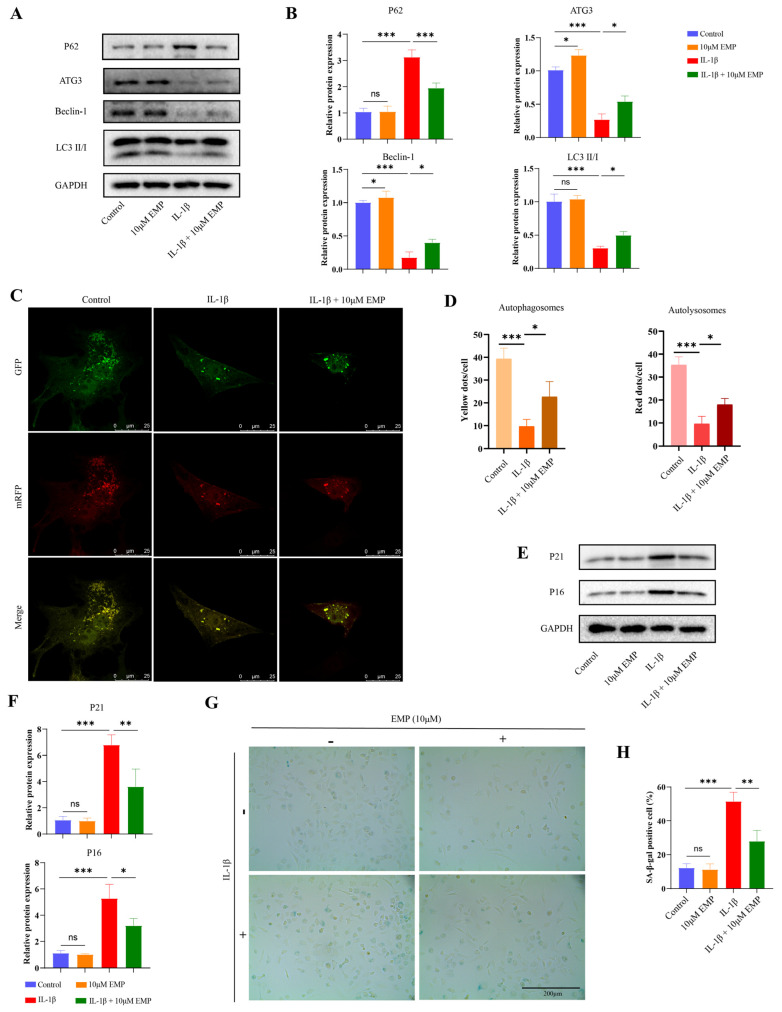
EMP restored the impaired autophagy and attenuated senescent phenotype in IL-1β treated chondrocytes. (**A**) The protein expression levels of P62, ATG3, Beclin-1, and LC3 II/I were analyzed by WB, and (**B**) quantitative analysis. (**C**) The strength of autophagic flux. (**D**) The quantitative analysis of autolysosomes and autophagosomes. (**E**) The protein expression levels of P21 and P16 were analyzed by WB, and quantitative analysis (**F**). (**G**) The SA-β-gal staining and quantitative analysis of SA-β-gal positive cell (**H**). Data are presented as mean ± SD (n = 3). Statistical significance was defined as *** *p* < 0.001, ** *p* < 0.01 and * *p* < 0.05. Non-significant differences were denoted as ns.

To elucidate the potential chondroprotective mechanism of EMP, the involvement of autophagic activity and senescence-associated phenotypes was assessed. Alterations in the protein expression level of autophagy related factors were first examined. WB analysis revealed that IL-1β significantly downregulated the protein expression levels of ATG3, beclin-1, and LC3 II/I, while upregulating P62 expression. In contrast, EMP treatment rescued the expression of autophagy related factors in IL-1β-treated chondrocytes (Figure 5A,B). Compared with the control group, IL-1β markedly reduced the total number of autophagic vesicles (yellow + red puncta), indicating impaired autophagic flux at the initial stage; EMP treatment increased the number of red and yellow puncta, indicating restored autophagic flux (Figure 5C,D).

As shown in Figure 5E,F, IL-1β markedly upregulated the protein expression level of P16 and P21. However, EMP treatment markedly downregulated the protein expression of senescent factors (P16 and P21) in IL-1β treated chondrocytes. This trend was further confirmed by SA-β-gal staining. As shown in Figure 5G,H, when compared with the IL-1β group, reduced percentage of SA-β-gal positive cell was found in IL-1β + EMP (10 μM) group.

### 3.6. Mechanism of EMP on OA Identified by Network Pharmacology and Experimental Validation

**Figure 6:**
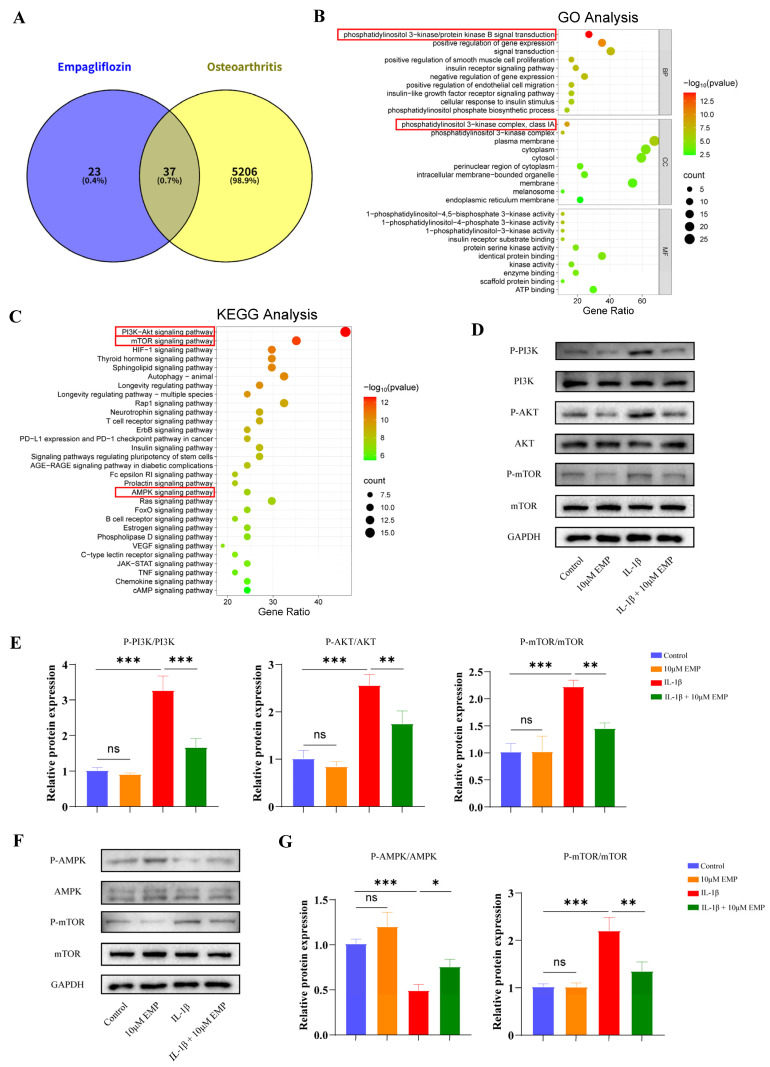
Mechanism of EMP on OA identified by network pharmacology and experimental validation. (**A**) Venn diagram of EMP and OA. (**B**) GO functional enrichment analysis. (**C**) Analysis of KEGG pathway enrichment. (**D**) WB analyzed the protein expression level of PI3K/Akt/mTOR pathway and (**E**) quantitative analysis. (**F**) WB analyzed the protein expression level of AMPK pathway and (**G**) quantitative analysis. Data are presented as mean ± SD (n = 3). Statistical significance was defined as *** *p* < 0.001, ** *p* < 0.01 and * *p* < 0.05. Non-significant differences were denoted as ns.

60 EMP-related targets were obtained and 5243 OA-related targets were obtained, respectively (Figure 6A). The GO analysis identified that the intersecting target genes were mainly involved in phosphatidylinositol 3-kinase/protein kinase B signal transduction in the terms of BP, and phosphatidylinositol 3-kinase complex, class IA in the terms of CC. The KEGG analysis identified that target genes were enriched in the PI3K-Akt, mTOR, and AMPK signaling pathways. These pathways were closely associated with autophagy [30,31,32], further indicating that the protective role of EMP on OA was mediated by regulating the autophagy process. WB analysis was employed to validate the results of network pharmacology. As shown in the Figure 6D,E, IL-1β treatment markedly activated the PI3K/Akt/mTOR signaling pathway, as evidenced by the elevated expression of P-PI3K/PI3K, P-Akt/Akt, and P-mTOR/mTOR, compared to the control group or EMP (10 μM) group. Whereas EMP treatment markedly reversed this trend in IL-1β treated chondrocytes. As shown in the Figure 6F,G, IL-1β treatment markedly inhibited the AMPK pathway, as evidenced by the reduced expression of P-AMPK/AMPK and the elevated expression of P-mTOR/mTOR, whereas EMP treatment also markedly reversed this trend. Notably, PI3K/Akt/mTOR inhibition and AMPK activation both downregulated the expression of P-mTOR/mTOR, resulting in the activation of autophagy process cooperatively.

### 3.7. The Role of Autophagy in EMP-Mediated Chondroprotection

**Figure 7:**
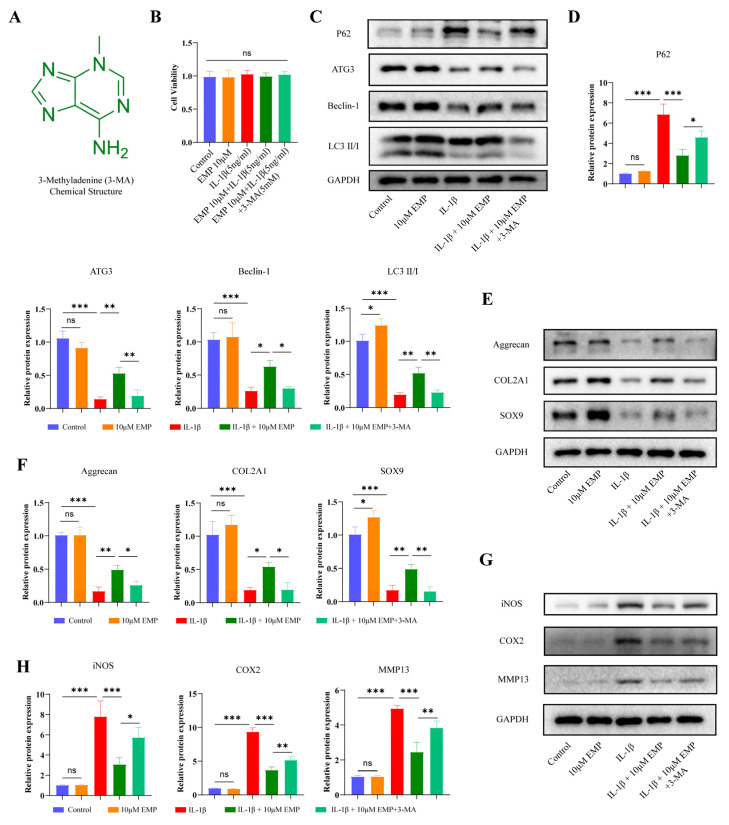
The autophagy-related effects of EMP were attenuated by 3-MA treatment. (**A**) The chemical structure of 3-MA. (**B**) The CCK8 analysis of chondrocytes viability treated with EMP or IL-1β or 3-MA. (**C**) The protein expression levels of P62, ATG3, Beclin-1, and LC3 II/I were analyzed by WB, and (**D**) quantitative analysis. (**E**) The protein expression levels of aggrecan, COL2A1 and SOX9 were analyzed by WB, and (**F**) quantitative analysis. (**G**) The protein expression levels of iNOS, COX2, and MMP13 were analyzed by WB, and quantitative analysis (**H**). Data are presented as mean ± SD (n = 3). Statistical significance was defined as *** *p* < 0.001, ** *p* < 0.01 and * *p* < 0.05. Non-significant differences were denoted as ns.

Our previous work identified that EMP could promote autophagy process in IL-1β treated chondrocytes and show an inhibitory effect on the PI3K/Akt/mTOR signaling pathway, 3-MA was further employed to validate this result. 3-MA was known to suppress autophagic flux and thus utilized as an autophagy inhibitor [33]. The chemical structure of 3-MA was shown in Figure 7A. The results of CCK8 indicated that 5 mM 3-MA did not exert any detectable impact on cellular viability. Therefore, a concentration of 5 mM was selected for subsequent experiments. As shown in the Figure 7C,D, 3-MA treatment significantly diminished the enhanced autophagy process induced by EMP, as evidenced by reduced expression of ATG3, beclin-1, and LC3 II/I, as well as the increased expression of P62 at the protein level. Concurrently, a marked attenuation of protective effect of EMP in IL-1β treated chondrocytes was observed. As shown in the Figure 7E,F, the protein expression of anabolic factors (aggrecan, COL2A1, and SOX9) were significantly reduced following the 3-MA treatment in IL-1β treated chondrocytes. In addition, the protein expression of catabolic and inflammatory factors, including iNOS, COX2 and MMP13, were significantly increased following the 3-MA treatment (Figure 7G,H). Collectively, these in vitro findings further indicated that the chondroprotective of EMP in IL-1β treated chondrocytes were mediated by regulating the autophagy process.

### 3.8. In Vivo Effects of EMP in DMM Models

**Figure 8:**
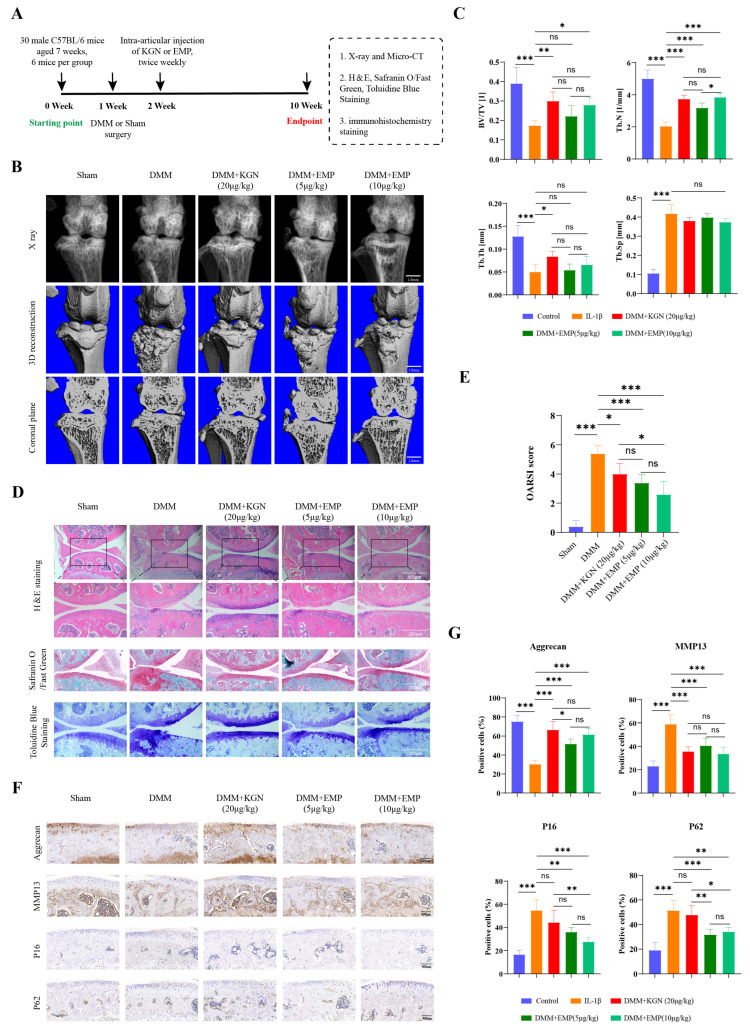
EMP ameliorated OA-related phenotype in DMM models. (**A**) The schematic diagram of the animal experiments. (**B**) Representative images of X-ray, 3D reconstruction, and coronal plane. (**C**) Quantitative analysis of key parameters in the tibial subchondral bone. (**D**) H&E staining, safranin O/fast green staining, and toluidine blue staining. (**E**) Quantitative analysis of the OARSI scores. (**F**) IHC staining of aggrecan, MMP13, P62, and P16, and (**G**) quantitative analysis of IHC staining positive cell. (n = 6). Statistical significance was defined as *** *p* < 0.001, ** *p* < 0.01 and * *p* < 0.05. Non-significant differences were denoted as ns.

An overview of the experimental design used for the animal studies was depicted in Figure 8A. As shown in the Figure 8B, when compared with the SHAM group, mice in the DMM group showed disrupted articular structure, including narrowed joint space and increased osteophyte formation, indicating the successful establishment of the OA models. Intra-articular injections of KGN (20 μg/kg) or EMP (5 μg/kg or 10 μg/kg) significantly alleviated the OA related changes in vivo induced by DMM surgery (Figure 8B). However, the results of X-ray and μ-CT showed no significant difference between the three groups. Moreover, quantitative analysis of the bone parameters showed that KGN (20 μg/kg) treatment significantly increased the reduced BV/TV, Tb.N and Tb.Th, EMP (5 μg/kg) treatment significantly increased the reduced Tb.N, and EMP (10 μg/kg) treatment significantly increased the reduced BV/TV and Tb.N (Figure 8C). Both KGN and EMP showed no significant effects on Tb.Sp (Figure 8C).

Histological and IHC staining were further employed to identify the changes in cartilage structure and protein expression profiles, including anabolic factor (aggrecan), catabolic factor (MMP13), senescence-associated factor (P16), and autophagy related factor (P62). As shown in the Figure 8D, the most severe cartilage destruction was observed in the DMM group. Intra-articular injections of KGN or EMP (5 μg/kg or 10 μg/kg) significantly alleviated the cartilage destruction in vivo when compared with the DMM group (Figure 8D). The chondroprotective effect of EMP (10 μg/kg) was found to be comparable to that of KGN (20 μg/kg). Accordingly, the protective effects of KGN or EMP (5 μg/kg or 10 μg/kg) were also validated by the OARSI scores (Figure 8E). Notably, the lowest OARSI score was recorded in the EMP (10 μg/kg) group. As expected, compared with the DMM group, mice in the DMM + KGN, DMM + EMP (5 μg/kg), DMM + EMP (10 μg/kg) showed upregulated expression of aggrecan and downregulated expression of MMP13. Moreover, intra-articular injections of EMP, particularly at a concentration of 10 μg/kg, significantly downregulated the expression of P16 and P62 (Figure 8F,G). However, KGN treatment shown no effect on the expression of P16 and P62. Notably, the dose-response relationship of EMP was not pronounced in vivo. Among all bone parameters, OARSI scores, and IHC staining, only Tb.N differed significantly between the two groups (5 μg/kg and 10 μg/kg).

Collectively, these in vivo data demonstrated that EMP alleviated the OA progression induced by DMM surgery. A more pronounced chondroprotective effect was observed with EMP treatment compared to KGN.

## 4. Discussion

**Figure 9:**
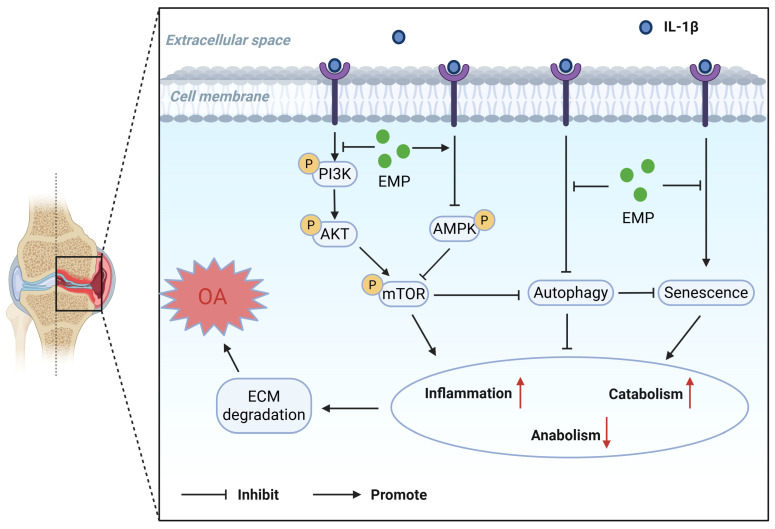
The schematic diagram illustrating the chondroprotective effect of EMP on murine OA. Created in BioRender. 1, 1. (2026) https://BioRender.com/nmhseyd (accessed on 1 April 2026).

To the best of our knowledge, this study is the first to report the chondroprotective role of EMP in OA. This study confirmed that: (1) EMP inhibited the inflammatory response and catabolism in IL-1β stimulated chondrocytes. (2) EMP promoted anabolism in IL-1β stimulated chondrocytes. (3) EMP restored the impaired autophagy and attenuated senescent phenotype in IL-1β treated chondrocytes. (4) EMP inhibited the PI3K/Akt/mTOR pathway and activated the AMPK pathway. (5) EMP ameliorated OA-related phenotype in DMM models. (6) EMP was found to exhibit a greater chondroprotective potential than KGN in vitro and in vivo. The schematic diagram illustrating the chondroprotective effect of EMP is presented in Figure 9.

A substantial number of antidiabetic drugs, such as sulfonylureas and metformin, were identified as disease-modifying agents for OA treatment [34,35,36,37]. As the most frequently prescribed agent for glycemic control, metformin was demonstrated to have a chondroprotective role both in clinical and experimental investigations. On the one hand, Zheng et al. [36] showed that metformin could suppress the inflammatory response of murine chondrocytes and DMM surgery induced cartilage degeneration by targeting synovial M1 macrophages. On the other hand, Pan et al. [37] has demonstrated the therapeutic effects of metformin in subjects with clinically diagnosed symptomatic knee OA through a randomized clinical trial. However, the role of SGLT2 inhibitors in OA remains largely unknown. In addition, a paradoxical relationship between SGLT2 inhibitors and OA has been suggested in two recent publications [34,38]. A retrospective cohort study indicated that SGLT2 inhibitors did not contribute to a significant reduction in OA risk when compared to metformin treatment [38]. Whereas the retrospective study design coupled with limited follow-up periods may result in the underestimation of its role. Fu et al. [34] suggested a potential causal relationship between SLC5A1 (the target of SGLT2 inhibitors) and OA phenotypes by employing the two-sample Mendelian Randomization, indicating that SGLT2 inhibitors may serve as a disease-modifying medication in OA. However, the results of Mendelian Randomization could be limited by confounders and the observational nature precluded definitive causal inferences. While these studies provide valuable epidemiological and genetic insights, direct experimental evidence for the effects of SGLT2 inhibitors on chondrocyte function and cartilage degradation in OA models remains lacking. Therefore, further studies should be conducted to explore and validate the role of SGLT2 inhibitors in OA. 

EMP, one of the SGLT2 inhibitors, is a clinical stage used, safe and effective oral hypoglycemic agent [15]. Published works indicated that EMP exhibited diverse biological properties, including anti-inflammatory, anti-senescence effect, and paradoxical role in autophagy [17]. In line with these results, our in vitro and in vivo findings first demonstrated that EMP also showed the potent anti-inflammatory and anti-senescence effects in chondrocytes, and thus alleviating osteoarthritic cartilage degradation. Similar to our findings, Zheng et al. [36] also reported the therapeutic effect of metformin, an anti-diabetic drug, on OA. However, their results indicated that metformin exerts its effect by regulating the PI3K/AKT pathway and downstream signaling to influence M1 macrophage polarization. In contrast, our study demonstrates that EMP affects chondrocyte metabolism, inflammation, and autophagy through the modulation of the PI3K/Akt/mTOR and AMPK signaling pathways. Besides, Zheng et al. [36] treated DMM induced mice with 100 or 200 mg/kg/d metformin by oral gavage. Whereas EMP was administered by intra-articular injection at a concentration of 5 or 10 μg/kg in our study, concurrently, a favorable therapeutic outcome was attained. Based on this, we held the opinion that the localized delivery of EMP by intra-articular injection proved highly effective for OA treatment in preclinical models.

A protective role of autophagy was demonstrated in the preservation of articular cartilage structure and function. Consistent with previous studies, impaired autophagy was observed both in IL-1β treated chondrocytes and DMM induced models in our study. However, in contrast to prior studies [23], the current data revealed that EMP showed an autophagy-promoting effect in OA. Treatment with EMP can restore the impaired autophagy in vivo and in vitro, as evidenced by the upregulated expression of positive factors (ATG3, beclin-1, and LC3 II/I), downregulated expression of negative factor (P62), and upregulated autophagic flux. However, these effects were significantly reversed by 3-MA treatment. Concurrently, alleviated phenotypes by EMP treatment in IL-1β treated chondrocytes were also reversed, strongly suggesting the involvement of autophagy in EMP therapeutic role. Moreover, to conduct a comprehensive evaluation of the effects of EMP on chondrocytes, KGN was selected as positive control in this study. Compared with KGN, EMP showed more significantly reduction in the expression of inflammatory (iNOS) and catabolic (MMP13) factors in vitro. Besides, KGN showed no effects on the expression of P16 and P62, whereas EMP significantly downregulated the expression of P16 and P62 in vivo. Collectively, the protective effects of EMP were superior than KGN both in vitro and in vivo. Therefore, the potential of EMP as a promising therapeutic option for OA was highlighted by these present findings.

Mechanistically, the PI3K/Akt/mTOR and AMPK signaling pathways were identified by the network pharmacological analysis and further experimentally studied and verified by WB. The AMPK signaling pathway has important role in tuning mitochondrial activity, regulating inflammation via inhibiting the NF-κB pathway, enhancing the autophagy process, and regulating the catabolism and anabolism in chondrocyte [14]. Clinically, some inflammatory conditions were routinely managed by AMPK activators, including well-established agents such as aspirin and metformin [14,39]. Moreover, Li et al. suggested that metformin could alleviate OA development by activating the AMPK signaling pathway in DMM surgery induced mice [35]. PI3K/Akt/mTOR pathway was also closely associated with inflammation, autophagy process, as well as cellular metabolism in OA [40]. Therefore, EMP may alleviate osteoarthritis progression by attenuating inflammation, restoring impaired autophagy, and ameliorating chondrocyte senescence through regulating the PI3K/Akt/mTOR and AMPK pathways.

Several strengths of this study need to be noted. Firstly, to the best of our knowledge, this study provides the first experimental evidence that EMP exerts chondroprotective effects in IL-1β treated chondrocytes and DMM induced models. Secondly, the multi-faceted effects of EMP on chondrocytes—including suppression of inflammation, enhancement of autophagic activity, and attenuation of cellular senescence—were consistently observed across both in vitro and in vivo models, supporting the robustness of these findings. Thirdly, an integrated approach combining network pharmacology with experimental validation identified the PI3K/Akt/mTOR and AMPK pathways as key mediators of EMP’s effects. Fourthly, when compared to KGN at the doses tested, EMP may exhibit a potentially greater protective effect, supporting the future clinical use of EMP. Fifth, given its extensive clinical history in diabetes management, low risk of side effects, and the improvement of cardiovascular and renal outcomes [41,42], EMP was repositioned as a candidate for OA treatment, offering enhanced convenience and safety.

Several limitations of the present investigation should also be considered. Firstly, our present work demonstrated that EMP could alleviate OA, but we did not elucidate whether other SGLT2 inhibitors, such as dapagliflozin and canagliflozin, have the same effect on OA. Secondly, the specific molecular mechanism has not been fully revealed. EMP is one of the SGLT2 inhibitors, however, the physiological effects of EMP were mediated through both SGLT2-dependent and SGLT2-independent pathways [43,44,45]. For example, the cardioprotective effect of EMP against heart failure was mediated via NHE1-NO pathway suppression, in an SGLT2-independent manner [43]. Our future studies will verify whether EMP’s protective effect against OA is SGLT2-dependent through SGLT2 overexpression or knockdown experiments. Whether and how the target (*SGLT2*) of EMP influence the OA onset and progression? Whether *SGLT2* overexpression or knockdown could alleviate or aggravate the OA progression in IL-1β treated chondrocytes and DMM induced models? Accordingly, our subsequent investigations were therefore focused on elucidating this particular mechanism. Thirdly, it is widely recognized that OA is a whole joint disease involving all joint tissues, and the pathology of OA involves multiple tissues or cell types [46,47,48]. However, we only evaluated the role of EMP on chondrocytes. Further studies evaluating the role of EMP on additional cell types, such as synovial cells, are still important. Finally, although we employed multiple complementary methods including LC3-II/I ratio, P62 degradation, and GFP-RFP-LC3 tandem fluorescent puncta analysis, the absence of lysosomal inhibitor controls (such as Bafilomycin A1) precludes definitive conclusions about whether EMP enhances autophagic flux by increasing autophagosome formation or by promoting autophagosome-lysosome fusion.

## 5. Conclusions

In conclusion, this preclinical study demonstrates that EMP can alleviate OA both in IL-1β stimulated chondrocytes and DMM induced models. Beyond its established role in diabetes management, EMP is evaluated in the context of OA, emerging as a promising therapeutic agent for OA. Future studies are still needed to elucidate the underlying mechanisms (SGLT2-dependent or SGLT2-independent pathways).

## Data Availability

The dataset supporting the conclusions of this article is included within the article.

## Supplementary Materials

The following supporting information can be downloaded at: https://www.mdpi.com/article/10.3390/biomedicines14040828/s1, Table S1 Primary antibodies for western blot; Table S2 The primer sequences used in the RT-qPCR experiment.

## Abbreviations

BSA	Bovine serum albumin
BV/TV	Bone volume/tissue volume
CCK-8	Cell counting kit-8
COX2	Cyclooxygenase 2
DMEM/F12	Dulbecco’s Modified Eagle Medium /Ham’s F 12
DMM	Destabilized medial meniscus
ECM	Extracellular matrix
EMP	Empagliflozin
FBS	Fetal bovine serum
IF	Immunofluorescence
IHC	Immunohistochemistry
iNOS	inducible nitric oxide synthase
KGN	Kartogenin
μ-CT	Micro-computed tomography
MMPs	Matrix metalloproteinases
OA	Osteoarthritis
OARSI	Osteoarthritis research society international
ROI	Region of interest
RT-qPCR	quantitative real-time polymerase chain reaction
SGLT2	Sodium-glucose cotransporter-2
Tb.N	Trabecular number
Tb.Sp	Trabecular separation
TBST	Tris-buffered Saline-Tween solution
Tb.Th	Trabecular thickness
WB	Western blot
3-MA	3-methyladenine
