# Supplementary material for: Empagliflozin Alleviates Osteoarthritis Progression by Attenuating Inflammation, Restoring Impaired Autophagy, and Ameliorating Chondrocyte Senescence
Source: Biomedicines. 2026 Apr 5;14(4):828. doi: 10.3390/biomedicines14040828 (PMC13113350; doi:10.3390/biomedicines14040828)
Supplement: Supplementary file 1 [file biomedicines-14-00828-s001.zip › biomedicines-4158900-supplementary.pdf]

Supplemental Table S1 Primary antibodies for western blot.

Antibodies	Source	Catalog number	Dilution ratio
GAPDH	Proteintech Group, Wuhan, China	60004-1-Ig	1:50000
Aggrecan	Proteintech Group, Wuhan, China	13880-1-AP	1:1,000
Collagen II	Proteintech Group, Wuhan, China	28459-1-AP	1:1,000
SOX9	Cell Signaling Technology, Beverly, MA, USA	#82630	1:1,000
INOS	Cell Signaling Technology, Beverly, MA, USA	#13120	1:1,000
MMP3	Proteintech Group, Wuhan, China	17873-1-AP	1:500
MMP13	Proteintech Group, Wuhan, China	18165-1-AP	1:1,000
COX-2	Cell Signaling Technology, Beverly, MA, USA	#12282	1:1,000
P-PI3K	Cell Signaling Technology, Beverly, MA, USA	#4228	1:1,000
PI3K	Cell Signaling Technology, Beverly, MA, USA	#4249	1:1,000
P-AKT	Proteintech Group, Wuhan, China	66444-1-Ig	1:2,000
AKT	Proteintech Group, Wuhan, China	60203-2-Ig	1:5,000
P-mTOR	Proteintech Group, Wuhan, China	67778-1-Ig	1:2,000
mTOR	Proteintech Group, Wuhan, China	66888-1-Ig	1:5,000
P-AMPKα	Proteintech Group, Wuhan, China	80209-6-RR	1:1,000
AMPKα	Proteintech Group, Wuhan, China	10929-2-AP	1:5,000
P62	Proteintech Group, Wuhan, China	18420-1-AP	1:5,000
Beclin-1	Cell Signaling Technology, Beverly, MA, USA	#3495	1:1,000
LC3II/I	Cell Signaling Technology, Beverly, MA, USA	#12741	1:1,000
ATG3	Cell Signaling Technology, Beverly, MA, USA	#3415	1:1,000

Supplemental Table S2 The primer sequences used in the RT-qPCR experiment.

Gene	Forward sequence (5'-3')	Reverse sequence (5'-3')
GAPDH	CCCAGCTTAGGTTCATCAGG	ATCTCCACTTTGCCACTGC
Aggrecan	CTCACCCCAAGAATCAAGTGG	GATCTCCAAGGTAGCATCGC
Collagen II	GGCCAGGATGCCCGAAAATTA	CGCACCCTTTTCTCCCTTGT
SOX9	AGCACTCTGGGCAATCTCA	GTTCACCGATGTCCACGTC
INOS	CTCCTGCCTCATGCCATTG	AGCTCATCCAGAGTGAGCTG
COX-2	GATAACCGAGTCGTTCTGCC	AATCCTGGTCGGTTTGATGC
MMP3	ACTCCCTGGGACTCTACCAC	GGTACCACGAGGACATCAGG
MMP13	TGATGGACCTTCTGGTCTTCTGG	CATCCACATGGTTGGGAAGTTCT
